# Altered gene expression in the superior temporal gyrus in schizophrenia

**DOI:** 10.1186/1471-2164-9-199

**Published:** 2008-04-29

**Authors:** Nikola A Bowden, Rodney J Scott, Paul A Tooney

**Affiliations:** 1 Neuroscience Institute of Schizophrenia and Allied Disorders, Sydney, NSW, Australia; 2 The Brain and Mental Health Research Program of the Hunter Medical Research Institute, John Hunter Hospital, Lookout Rd, New Lambton Heights, NSW Australia; 3 School of Biomedical Sciences, University of Newcastle, Callaghan 2308, Australia; 4 Division of Genetics, Hunter Area Pathology Service, John Hunter Hospital, Lookout Rd, New Lambton Heights 2305, Australia

## Abstract

**Background:**

The superior temporal gyrus (STG), which encompasses the primary auditory cortex, is believed to be a major anatomical substrate for speech, language and communication. The STG connects to the limbic system (hippocampus and amygdala), the thalamus and neocortical association areas in the prefrontal cortex, all of which have been implicated in schizophrenia.

**Results:**

To identify altered mRNA expression in the superior temporal gyrus (STG) in schizophrenia, oligonucleotide microarrays were used with RNA from postmortem STG tissue from 7 individuals with schizophrenia and 7 matched non-psychiatric controls. Overall, there was a trend towards down-regulation in gene expression, and altered expression of genes involved in neurotransmission, neurodevelopment, and presynaptic function was identified. To confirm altered expression identified by microarray analysis, the mRNA expression levels of four genes, IPLA2γ, PIK31R1, Lin-7b and ATBF1, were semi-quantitatively measured using relative real-time PCR. A number of genes with altered expression in the STG were also shown to have similar changes in expression as shown in our previous study of peripheral blood lymphocytes in schizophrenia.

**Conclusion:**

This study has identified altered expression of genes in the STG involved in neurotransmission and neurodevelopment, and to a lesser extent presynaptic function, which further support the notion of these functions playing an integral role in the development of schizophrenia.

## Background

The introduction of cDNA microarrays has identified changes in the expression of hundreds of genes in post-mortem brain tissue from individuals with schizophrenia [1-14]. These studies have identified new genes with both altered expression in, and genetic association to schizophrenia. Many functional groups of genes have been reported to be altered in these studies including those involved in neurotransmission, presynaptic functioning, myelination, neurodevelopment and basic cellular processes such as cell-cycle regulation and intracellular signalling. The most studied region for gene expression analysis is the prefrontal cortex (PFC), with fewer comprehensive gene expression profiling studies reported for other cortical regions.

The superior temporal gyrus (STG), which encompasses the primary auditory cortex and is believed to be a major anatomical substrate for speech, language and communication [15]. The STG connects to the limbic system (hippocampus and amygdala), the thalamus and neocortical association areas in the prefrontal cortex, all of which have been implicated in schizophrenia. Significant evidence suggests the STG plays an important role in the pathophysiology of some symptoms of schizophrenia. Structural MRI studies have consistently reported the left STG volume to be decreased in patients with schizophrenia compared to healthy controls [16-20], which was confirmed by a recent meta-analysis of 15 voxel-based morphometry studies [21]. Other studies have reported a correlation between STG volume and the severity of auditory hallucinations [22,23] and thought disorder [15]. In addition, the STG is considered to be the generator of mismatch negativity (MMN), an auditory phenomenon, which is reduced in individuals with schizophrenia [24,25].

There are limited reports of post mortem studies of the cytoarchitecture in the temporal cortex in schizophrenia. A reduction in the somal volume of deep layer 3 pyramidal cells in the temporal association cortex has been reported [26], however, [27] and colleagues reported no alteration of glial density or neuronal size and density in the Planum Temporale (PT), the posterior portion of the STG, in schizophrenia. With respect to neurotransmission, decreased density of muscarinic receptors [28] and increased density of GABA_A _receptors [29] have been reported in the STG. Despite evidence indicating functional disturbances of the auditory cortex and connections to most of the brain regions implicated in schizophrenia, the STG has to date been largely overlooked in large-scale gene expression, cellular composition and functional studies in schizophrenia.

In this study, oligonucleotide microarrays were used to identify altered gene expression in 7 age, gender, PMI and pH matched pairs of post-mortem STG tissue from individuals with schizophrenia and healthy controls. To confirm altered expression of 4 genes, an additional 6 matched pairs (n = 13) were included in relative real-time PCR confirmation studies. The expression of two previously reported putative genes for schizophrenia, RGS4 [30,31] and RIMS2 [14] was also measured. In addition, the genes found to be significantly altered in the STG were compared to those previously reported to be altered in peripheral blood lymphocytes (PBLs) [32]. The findings of this study suggest that gene expression changes in the STG may be important in the pathophysiology of this disorder.

## Results

### Altered gene expression in the STG from microarrays

**Figure 1:**
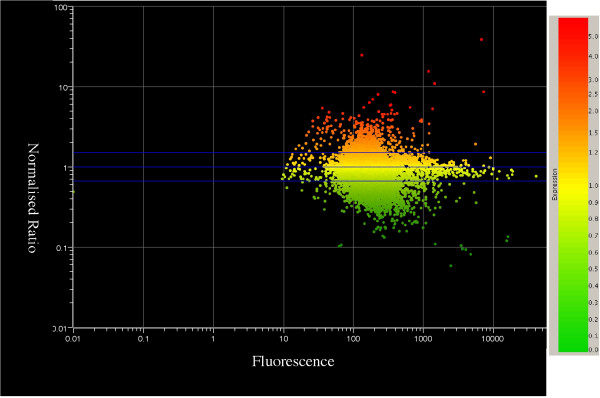
**Average ratio of expression of 19,000 genes in the STG from participants with schizophrenia compared to non-psychiatric controls**. The central blue line indicates an expression ratio of 1 (ie: equal expression) and the two outer blue lines indicate a 1.5-fold change. Down-regulation is represented as green, up-regulation as red and normal expression as yellow. 191 genes were up-regulated by more than 1.5 fold and 428 were down-regulated by greater than 1.5 fold. The x-axis shows the average level of fluorescence present on the microarrays for each gene.

An individual gene was considered to be expressed if fluorescence was detected above background levels in at least 4 of the 7 matched pairs. This criterion identified 8737 genes as being expressed in the STG tissue. The average gene expression for all 19,000 genes across all 7 matched pairs was calculated and plotted against the level of fluorescence detected (Figure 1). The scatter plot depicts a slight trend towards overall down-regulation in individuals with schizophrenia compared to healthy controls. On average, the expression of 2448 genes were down-regulated and 946 genes up-regulated, by 1.5 fold or greater in the STG in schizophrenia compared to controls. To further define this list, genes with altered expression greater than 1.5 fold present in more than 50% (4/7) of the cohort were selected for further analysis. Using this criteria 191 genes were up-regulated by more than 1.5 fold and 428 were down-regulated by greater than 1.5 fold in the STG in schizophrenia.

**Figure 2:**
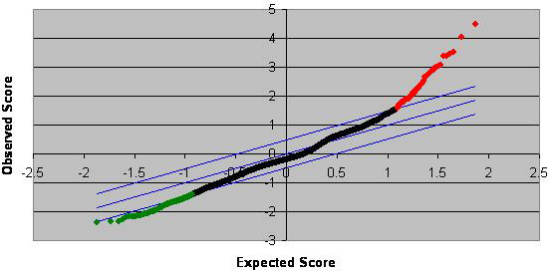
**SAM plot of gene expression in the STG from participants with schizophrenia compared to non-psychiatric controls**. Average expression of 8737 genes expressed in STG tissue from 7 matched pairs of individuals with schizophrenia and non-psychiatric controls. The central blue line indicates equal expression and the two outer blue lines indicate significantly altered expression (Δ = 0.49, FDR = 0.47), genes in red were significantly up-regulated in schizophrenia compared to controls and genes in green were significantly down-regulated in schizophrenia compared to controls. 216 genes were significantly down-regulated and 85 genes were significantly up-regulated.

One-class analysis of log 2 transformed expression data present in at least 4 of the 7 matched pairs was performed using SAM version 2.0 [33] with 128 permutations. This limited the list of genes altered by greater than 1.5 fold to only those genes with significantly altered expression and as such identified 216 significantly down-regulated genes and 85 significantly up-regulated genes (Δ = 0.49) with a false discovery rate (FDR) of 4.7% (Figure 2).

Some of the genes that were significantly altered in the STG are located within the most reproduced schizophrenia linkage loci (Table 2) as were genes with functions known to be associated with schizophrenia such as neurotransmission, myelination and neurodevelopment (Table 3).

**Table 1:** Demographic data for matched pairs consisting of individuals with schizophrenia and non-psychiatric controls.

Pair	Gender	Age (years) (sz/cntrl)	Medication (CPE) (mg/day)	Duration of Illness (years)	Frozen Hemisphere	Cause of Death (sz/cntrl)	PMI (hours) (sz/cntrl)	pH (sz/cntrl)
1	M	51/50	100	24	L	ischaemic heart disease/ischaemic heart disease	21/19	6.02/6.26
2	M	57/58	unknown	26	L	cardiac arrhythmia/ischaemic heart disease	33/38	6.4/6.5
3	M	52/59	450	31	R	ischaemic heart disease/coronary thrombosis	8/20	6.1/6.56
4	M	44/43	500	17	L	Suicide/thrombotic coronary artery occlusion	35/13	6.55/6.43
5	M	30/34	130–520	3.5	L	Suicide/acute exacerbation of asthma	24/20.5	6.6/6.73
6	M	32/38	780	13	L	Suicide/atherosclerotic cardiovascular disease	25/13.5	6.24/6.26
7	M	51/46	300–1300	30	R	ischaemic heart disease/cardiac arrest	18/25	6.62/6.7
8	F	67/70	150–1100	46	R	Emphema/ischaemic heart disease	27/30	6.2/6.8
9	M	75/73	200–1200	44	L	ischaemic heart disease/respiratory arrest	36/10	6.4/6.2
10	M	54/56	50–600	35	R	Coronary artery thrombosis/cardiac arrest	27/37	6.2/6.8
11	F	61/56	800–1500	42	R	ischaemic heart disease/pulmonary thromboembolus	49/23	6.7/6.7
12	M	67/69	200–2400	41	R	ischaemic heart disease/cardiovascular disease	5/16	6.4/6.6
13	M	57/56	225–975	17	R	cardiovascular disease/Cardiac atheroma	48/24	6.7/6.5

Means SZ Control		53.7 ± 13.0 54.5 ± 12.1	*637.9 ± 342.7	28.4 ± 13.2			27.4 ± 13.2 22.2 ± 8.7	6.4 ± 0.2 6.5 ± 0.2

*calculated using the mean CPE dosage for each matched pair.

**Table 2:** Genes in schizophrenia linkage sites with significantly altered expression in the STG in schizophrenia.

**SZ Linkage Site**	**GenBank ID**	**Name**	**Symbol**	**Cytoband**	**# pairs changed/7**	**Average fold change**
1q21-q22	NM_006271	S100 calcium binding protein A1	S100A1	1q21	5	-1.70
	NM_014624	S100 calcium binding protein A6 (calcyclin)	S100A6	1q21	4	-2.36
	J03651	Mucin 1, transmembrane	MUC1	1q21	4	-1.94
	AF131738	Hypothetical LOC440675		1q21.1	6	2.05
	AJ009640	Sorting nexin family member 27	SNX27	1q21.3	6	-2.13
	AK001560	Immunoglobulin superfamily, member 4B	IGSF4B	1q21.2-q22	4	-2.19
	NM_014328	RUN and SH3 domain containing 1	RUSC1	1q21-q22	5	-3.03
	AB040968	Hyperpolarization activated cyclic nucleotide-gated potassium channel 3	HCN3	1q22	5	1.95
1q32-q42	AK024293	Hypothetical gene AK024293	AK024293	1q32.1	4	1.82
	AB037804	KIAA1383	KIAA1383	1q42.2	6	4.09
5q21-q34	NM_014945	Actin binding LIM protein family, member 3	ABLIM3	5q32	4	-2.29
	AB032364	Early B-cell factor	EBF	5q34	5	1.91
6p24-p21	AJ277276	Transcriptional regulating factor 1	TRERF1	6p21.1-p12.1	4	-2.38
	AB033072	Leucine rich repeat and fibronectin type III domain containing 2	LRFN2	6p21.2-p21.1	4	-2.38
	AB002379	Dishevelled associated activator of morphogenesis 2	DAAM2	6p21.2	4	1.69
	AF024699	Zinc finger protein 193	ZNF193	6p21.3	6	3.73
	NM_014260	HLA class II region expressed gene KE2	HKE2	6p21.3	4	-2.18
	AK024858	LEM domain containing 2	LEMD2	6p21.31	4	-2.27
	AF085841	Proline-rich transmembrane protein 1	C6orf31	6p21.32	5	-2.47
	AK026291	Ribonuclease P 21 kDa subunit	RPP21	6p21.33	5	-2.17
	NM_003520	Histone 1, H2bn	HIST1H2BN	6p22-p21.3	6	5.01
	AK023284	Testis expressed sequence 27	TEX27	6pter-p22.3	7	4.17
6q12-q26	AK026849	Squamous cell carcinoma antigen recognized by T cells 2	TSPYL1	6q22-q23	4	-3.79
	AK026490	RAB32, member RAS oncogene family	RAB32	6q24.3	5	-1.91
8p22-p21	NM_002027	Farnesyltransferase, CAAX box, alpha	FNTA	8p22-q11	4	1.72
	NM_015458	Myotubularin related protein 9	MTMR9	8p23-p22	4	-2.21
13q13-q32	AF097645	DEAD/H (Asp-Glu-Ala-Asp/His) box polypeptide 26	DDX26	13q14.12-q14.2	6	3.49
22q11-q13	AK022886	Activating signal cointegrator 1 complex subunit 2	ASCC2	22q12.1	5	1.96
	NM_000967	Ribosomal protein L3	RPL3	22q13	5	-3.65
	NM_002305	Lectin, galactoside-binding, soluble, 1 (galectin 1)	LGALS1	22q13.1	4	-3.11
	AK025423	Hypothetical protein MGC52010	MGC52010	22q13.1	5	-1.67
	NM_002676	Phosphomannomutase 1	PMM1	22q13.2	6	-2.52
	NM_002490	NADH dehydrogenase (ubiquinone) 1 alpha subcomplex, 6	NDUFA6	22q13.2-q13.31	5	-2.83
	AK026892	Ceramide kinase	CERK	22q13.31	5	-1.83

**Table 3:** Genes with significantly altered expression in the STG in schizophrenia according to functional group.

**Functional Group**	**GenBank ID**	**Name**	**Symbol**	**Cytoband**	**# pairs changed/7**	**Average Fold Change**
Neuro- transmission	NM_000834	Glutamate receptor, ionotropic, N-methyl D-aspartate 2B	GRIN2B	12p12	4	1.98
	AL157442	Glutamate receptor, ionotropic, N-methyl D-asparate-associated protein 1 (glutamate binding)	GRINA	8q24.3	6	-2.33
	AK024507	Glutamate receptor interacting protein 2	GRIP2	3p24-p23	4	-1.73
	AB043997	Solute carrier family 5 (choline transporter), member 7	SLC5A7	2q12	5	2.07
	AF263613	Intracellular membrane-associated calcium-independent phospholipase A2 gamma*	IPLA2(γ)	7q31	6	1.88
	AF311862	Lin-7 homolog B (C. elegans)	LIN7B	19q13.3	6	-3.00
	AL137559	Synaptotagmin VII	SYT7	11q12-q13.1	4	-1.90
	NM_007232	Histamine receptor H3	HRH3	20q13.33	4	-2.00
Presynaptic function	AJ132089	Dynein, axonemal, heavy polypeptide 10	DNAH10	12q24.31	6	4.08
	AB011131	Piccolo*	PCLO	7q11.23-q21.3	4	-2.23
Myelination	AF179481	Acyl-CoA synthetase bubblegum family member 1*	Ascbg1	15q23-q24	5	-1.94
Neuro- development	AL050059	Disabled homolog 1 (Drosophila)	DAB1	1p32-p31	4	-2.49
	NM_006885	AT-binding transcription factor 1	ATBF1	16q22.3-q23.1	6	2.77
	NM_006156	Neural precursor cell expressed, developmentally down-regulated 8	NEDD8	14q11.2	5	-2.62
	AF087991	Netrin G1	NTNG1	1p13.3	5	-2.57
	AF009311	Semaphorin 5A	SEMA5A	5p15.2	4	2.23
Intracellular signalling	M61906	Phosphoinositide-3-kinase, regulatory subunit 1 (p85 alpha)	PIK3R1	5q13.1	7	4.10
	NM_003632	Contactin associated protein 1	CNTNAP1	17q21	4	-2.74
	NM_006078	Calcium channel, voltage-dependent, gamma subunit 2	CACNG2	22q13.1	4	-2.16
	NM_003385	Visinin-like 1	VSNL1	2p24.3	5	-2.15
	NM_005031	FXYD domain containing ion transport regulator 1 (phospholemman)*	FXYD1	19q13.1	5	-3.07
Other brain related illnesses	NM_005503	Amyloid beta (A4) precursor protein-binding, family A, member 2 (X11-like)	APBA2	15q11-q12	4	-2.55
	NM_004860	Fragile × mental retardation, autosomal homolog 2	FXR2	17p13.1	5	-2.16
Other functions	AB002340	Lupus brain antigen 1	LBA1	3p22.3	4	-1.89
	NM_002629	Phosphoglycerate mutase 1 (brain)	PGAM1	10q25.3	4	-2.18
	NM_016223	Protein kinase C and casein kinase substrate in neurons 3	PACSIN3	11p12-p11.12	5	-2.22
	NM_005619	Reticulon 2	RTN2	19q13.32	4	-1.72
	NM_001048	Somatostatin	SST	3q28	5	-3.02
	U27768	Regulator of G-protein signalling 4*	RGS4	1q23.3	4	-1.79
	M96577	E2F transcription factor 1	E2F1	20q11.2	6	-2.59
	AK023573	Synapse defective 1, Rho GTPase, homolog 1 (C. elegans)	7h3	19p13.12	4	-1.71
	X87825	Olfactory receptor, family 7, subfamily E, member 47 pseudogene	OR7E47P	12q13.13	6	7.32
	X89668	Olfactory receptor, family 7, subfamily E, member 19 pseudogene	OR7E19P	19p13.2	7	2.23

*Previously reported to be altered in schizophrenia.

### Relative real-time PCR confirmation of altered expression

Relative real-time PCR was used to confirm the altered expression of 4 genes with functional relevance to schizophrenia. Phosphoinositide-3-kinse regulatory subunit polypeptide 1 (PIK3R1), AT-binding transcription factor 1 (ATBF1), Lin-7 homolog b (Lin-7b) and calcium-independent phospholipase A2 gamma (IPLA2γ), had significantly altered expression in the STG in schizophrenia identified by microarray analysis. In addition to the 7 matched pairs used for the microarray analysis, 6 extra matched pairs were added to enlarge the cohort to 13 matched pairs. Relative real-time PCR analysis confirmed the microarray data for two of the genes, identifying significant up-regulation of ATBF1 (p = 0.01, df = 12, t = 2.56) and IPLA2G (p = 0.04, df = 12, t = 1.94) by 2.39 and 1.53 fold respectively. Although, relative real-time PCR confirmed the trends in expression from the microarray analysis with a 1.24 fold up-regulation of PIK31R1 and 1.29 fold down-regulation of Lin7b, these fold changes were not significant (PIK31R1: p = 0.29, df = 12, t = 0.57; Lin7b: p = 0.23, df = 12, t = -0.76) (Table 4).

**Table 4:** Fold changes in gene expression in the STG from individuals with schizophrenia compared to non-psychiatric controls that were identified by microarrays and confirmed by relative real-time PCR.

		**Microarray results**		**Real-Time PCR results**		
**GenBank ID**	**Symbol**	**Fold Change**	**Pairs Changed/7**	**Fold Change**	**Pairs changed/13**	**P Value**
NM_006885	ATBF1	2.77	6	2.39	11	0.01
AF263613	IPLA2G	1.88	6	1.53	9	0.04
M61906	PIK31R1	4.1	7	1.24	9	0.29
AF311862	Lin7b	-3	6	-1.29	9	0.39

### Expression levels of putative schizophrenia genes using relative real-time PCR

The expression of a number of putative schizophrenia candidate genes was analysed using relative real-time PCR in the STG in the subjects with schizophrenia and compared to controls. RGS4 was present on the microarrays and displayed a 1.79 fold down-regulation which was significant by SAM analysis. The average expression level of RGS4 mRNA in this cohort measured by relative real-time PCR was previously reported [31] to be down-regulated by -2.06 fold (p = 0.03, df = 12, t = -2.33) in the schizophrenia subjects compared to the controls and there was no significant correlation of altered RGS4 expression and age (R^2 ^= 0.27, p = 0.18), brain pH (R^2 ^= 0.20, p = 0.32) or PMI (R^2 ^= 0.15, p = 0.46). The decreased level of expression of RGS4 mRNA was present in 10 of the 13 matched pairs [31].

In addition, the RIMS2 mRNA expression level in the STG, a cytomatrix active zone gene, was investigated since it had been previously reported to be up-regulated in the amygdala [14]. Relative real-time PCR analysis of the mRNA expression levels of RIMS2 was performed and detected in 12 of the 13 matched pairs using primers and real-time PCR conditions for RIMS2 as previously described [14]. The control STG from pair 1 did not express RIMS2 at a detectable level in both microarray and real-time PCR analysis and so was excluded. The average expression level of RIMS2 mRNA detected by relative real-time PCR in the 12 matched pairs and normalized to 18s rRNA, was 1.56 × 10^-4 ^in schizophrenia and 9.09 × 10^-5 ^in the non-psychiatric controls. The resultant average fold change in expression of RIMS2 mRNA in the schizophrenia subjects compared to the controls was up-regulated by 1.72 fold (p = 0.03, df = 11, t = 2.15) and there was no significant correlation of altered RIMS2 expression and age (R^2 ^= -0.07, p = 0.74), brain pH (R^2 ^= -0.19, p = 0.36) or PMI (R^2 ^= -0.11, p = 0.61). The up-regulation of RIMS2 mRNA was present in 9 of the 12 matched pairs. Pair 7, 12 and 13 displayed down-regulation of RIMS2 by 1.37, 1.52 and 2.56 fold, respectively. Although, RGS4 and RIMS2 were altered in 10 of 13 pairs and 9 of 12 pairs respectively, the pairs which did not have altered expression of RGS4 were not the same pairs as those which did not have altered RIMS2.

### Consistent altered expression in PBLs and post-mortem STG

The data obtained for gene expression in the STG in schizophrenia was then compared to gene expression data generated using microarrays in a previous study of PBLs from individuals with schizophrenia compared to controls [32]. None of the cases or controls were the same in each study. However, three genes were shown to have similar altered expression in the PBLs and post-mortem STG tissue from individuals with schizophrenia, when compared to non-psychiatric controls using microarray analysis (Table 5). Myotubularin-related protein 9 (MTMR9) and Nuclear Factor κβα (NFκβα) were significantly down-regulated by SAM analysis in the STG and by greater than 1.5 fold in the PBLs in more than 7 of the matched pairs. Ewing Sarcoma breakpoint region 1 (EWRS1) was significantly up-regulated by SAM analysis in the STG and up-regulated by greater than 1.5 fold in the PBLs of 8 or more of the matched pairs.

**Table 5:** Genes with altered expression in post-mortem STG and PBLs from individuals with schizophrenia.

GenBank Acc.	Name	STG Pairs changed/7	Average STG fold change	PBLs Pairs changed/14	Average PBLs fold change
NM_015458	MTMR9	5	-2.2	11	-2.2
NM_020529	NFκβα	6	-2.8	10	-1.3
NM_005243	EWSR1	4	1.5	7	1.15

## Discussion

The use of high throughput tools such as microarrays has broadened the understanding of gene expression alterations in several brain regions in major mental illnesses such as schizophrenia. These regions include the PFC [1-4,6,8,12,13,30], cerebellum [9], amygdala [14], hippocampus [10], as well as the cingulate [13], temporal [9,11,13], parietal [13], enterohinal [7] and occipital [13] cortices. Interestingly, the STG, a region reported to be dysfunctional in schizophrenia has the most pronounced changes in gene expression when compared to most of the other regions implicated in schizophrenia [13].

The number of genes expressed in the cerebral cortex has been largely unreported in previous gene expression studies of the PFC, temporal, parietal and occipital cortices. Although significantly altered gene expression has been reported for these areas, identifying the approximate number of genes expressed, and identifying similar expression patterns across regions, could lead to furthering our understanding of the functional and biological similarities and differences between cortical areas. In this study, 8737 of the 19000 genes tested were detectable in the STG in 4 or more of the 7 matched pairs studied, this is in contrast to our study of the amygdala, where only 5394 genes were detected using the same 19000 gene microarray [14]. 4667 genes were expressed in both the amygdala and STG. Although the STG connects with the amygdala, the functions of the two regions are quite distinct which may be reflected in the difference in numbers of genes expressed. Mirnics and colleagues (2000) reported an average of 3735 genes expressed in the PFC of 9 individuals with schizophrenia, which is less than half that for the STG. However the microarray used by Mirnics and colleagues (2000) contained 7000 gene transcripts and the criteria to define genes that are expressed different from this study. Therefore, if tested using a larger gene set (e.g. 19000) the number of genes expressed in the PFC may be much higher.

One of the most consistent results across post-mortem brain studies is a trend towards overall down-regulation of genes in schizophrenia when compared to healthy controls [4,9,13,14]. The average expression of the gene transcripts measured in this study showed an overall trend towards down-regulation in the 7 matched pairs of STG tissue which was similar to that previously reported for other brain regions in schizophrenia. This global down-regulation across many brain regions, particularly in the cerebral cortex including the STG, may contribute to the generalized deficiencies seen in individuals with schizophrenia, such as poor performance on cognitive tasks, reduced MMN, pre-pulse inhibition (PPI) and eye-tracking dysfunction. Although the biological cause underpinning these deficiencies is yet to be identified, overall down-regulation of gene expression in many brain regions may be the first steps towards explaining these phenomena.

The number of genes with altered expression in the STG identified by SAM and a 1.5 fold change cut-off in this study was remarkably similar to that reported by Katsel and colleagues (2005) using a high stringency criteria. This study identified 216 genes down-regulated and 85 genes up-regulated whereas Katsel and colleagues (2005) reported 185 genes down-regulated and 88 genes up-regulated in the same region in 22 schizophrenia and control samples. Although the genes identified in each study could not be directly compared.

The dysregulation of gene expression observed in the STG also highlighted a number of key cellular pathways including neurotransmission, particularly glutamate signaling, neurodevelopment and neuronal differentiation and presynaptic function. Indeed, there is building evidence for dysfunction of glutamate synapses in the STG and temporal lobe in general. For example, increased density of the N-methyl-D-aspartate (NMDA) glutamate receptor was reported by Nudmamud and colleagues [34] using ligand binding studies and Le Corre and colleagues [35] used *in situ *hybridization to show increased density of the NMDA NR1 subunit splice variant in the STG from subjects with schizophrenia. In addition, Eastwood and Harrison (2001) [36] reported that complexin II mRNA (expressed in excitatory neurons) was reduced in the dorsolateral PFC and the superior temporal cortex, and complexin I mRNA (expressed in inhibitory neurons) was decreased in the superior temporal cortex in schizophrenia. Furthermore, several of the schizophrenia candidate genes (e.g. glutamate receptor, metabotropic 3 (GRM3), G72, D-amino acid oxidase (DAAO), proline dehydrogenase oxidase 1 (PRODH), neuregulin 1 (NRG1) and protein phosphatase 3 catalytic subunit gamma isoform (PPP3CC)) are thought to be directly or indirectly linked with glutamatergic transmission via NMDA receptors [37,38].

In this current study of the STG, the IPLA2γ gene was significantly up-regulated. IPLA2γ is an isoform of IPLA2 expressed in many tissue types, including the brain [39]. IPLA2, a key enzyme for phospholipid degradation, is vital for the maintenance and formation of cellular membranes [40]. IPLA2 is involved in neurotransmission via modulation of phosphorylation of the AMPA glutamate receptors. Endogenous IPLA2 activity limits phosphorylation on serine sites of the AMPA receptor GluR1 subunit, resulting in IPLA2 control over AMPA mediated synaptic transmission in the hippocampus [41,42]. There are, however, limited reports of IPLA2 function in other brain regions and the control over AMPA-mediated transmission is yet to be studied in areas such as the STG. IPLA2 is thought to be involved in learning and memory via alterations in hippocampal plasticity [43], deficiencies of which are key indicators of schizophrenia. Contrary to most reports being related to hippocampal tissue, IPLA2 protein expression has previously been reported to be increased by up to 45% in post-mortem temporal cortex [44] and in blood serum of individuals with schizophrenia [45,46], thus suggesting IPLA2 is worthy of further study.

Furthermore, the microarray analysis also showed that other genes involved in glutamate signaling were altered in the STG in patients with schizophrenia. These include, glutamate receptor, ionotropic, N-methyl D-aspartate 2B (GRIN2B) subunit, also known as NR2B, that had significantly up-regulated expression, and two glutamate receptor interacting proteins glutamate receptor N-methyl D-asparate-associated protein 1 (GRINA) and glutamate receptor interacting protein 2 (GRIP2) showing significant down-regulated expression in schizophrenia subjects. Furthermore, Lin-7b, which was downregulated in the STG of schizophrenia patients by microarray analysis, is highly enriched in post-synaptic densities (PSD) in association with PSD95/NMDA receptor complexes [47]. More specifically, Lin-7b is a PSD-95/Dlg/ZO-1 (PDZ) domain containing protein, which increases the channel activity of the NR1-NR2B glutamate receptor [48]. Thus, the evidence from this study supports dysfunction of glutamate transmission in the STG in the pathophysiology of schizophrenia.

Alteration to neurodevelopment and neuronal differentiation in the STG is supported by the significant up-regulation of ATBF1 by microarray and real-time PCR analysis in the STG tissue from individuals with schizophrenia compared to the non-psychiatric controls. This gene is highly expressed in the central nervous system, in particular dopaminergic neurons during neuronal differentiation [49]. The expression of ATBF1 results in suppression of the nestin gene and activation of the Neurogenic differentiation 1 (NEUROD1) gene, which are responsible for specific neuronal differentiation [50]. NEUROD1 was up-regulated in a gene expression study of the middle temporal gyrus (MTG) in schizophrenia [11], which may be a direct result of ATBF1 up-regulation similar to that observed in this study. Overexpression of ATBF1 in neuroblastoma cell lines produces cell cycle arrest, which is thought to occur in vivo as the result of nuclear localization of ATBF1 in differentiating neurons [50]. Therefore, overexpression of ATBF1 in the STG of patients with schizophrenia might reflect deficits in neuronal differentiation and neurodevelopment

There have been a number of reports indicating dysregulation of presynaptic function genes [51]. Indeed, our previous study of the amygdala [14] highlighted dysfunction of the genes coding for proteins in the cytomatrix active zone, a specialized region of the synapse involved in the regulation of vesicle release. RIMS2 codes for a protein that forms part of the cytomatrix of the active zone of synapses and was observed to be upregulated in the amygdala in schizophrenia [14]. The STG directly connects to the limbic system, of which the amygdala is a constituent. In this study RIMS2 was significantly up-regulated by 1.72 fold, similar to the 2.37 fold change reported in the amygdala. In contrast, Mirnics et al (2001a) reported a downregulation of presynaptic function genes, although those genes were not investigated in this study. RIMS2 modulates Ca^2+^-triggered exocytosis and may be overexpressed in the STG as a compensatory mechanism to overcome other presynaptic function deficiencies.

Regulator of G-protein signaling 4 (RGS4) is a schizophrenia candidate gene that was identified by Mirnics and colleagues [30,51] and has subsequently been reported in linkage [52] and convergent functional genomics studies [53]. The relative expression level of RGS4 mRNA in the STG from the cohort used in this study has been previously reported as significantly down-regulated by an average fold change of -1.93 by relative real-time PCR [31], which was confirmed in the current study as a significant down-regulation of -1.79 fold by microarray analysis. This down-regulation of RGS4 expression in the STG is in accordance with the decreased expression previously reported in the PFC, motor and visual cortices in schizophrenia [30] further establishing RGS4 as a schizophrenia candidate gene.

Changes in gene expression in PBLs are emerging as biological reflections of altered expression in the brain as described in [32]. Consistent with previous studies [54] 3 genes, MTMR9, EWSR1 and NFκβα were altered in the same direction in the post-mortem STG tissue and the peripheral blood lymphocytes (PBLs) from a separate cohort of individuals with schizophrenia identified in a previous study [32]. Whether this may lead to the use of PBLs in the development of a biological basis for identifying individuals with schizophrenia awaits further investigation.

The findings of this study and previous gene expression studies involving post-mortem brain tissue are limited by the availability of tissue and the potential of mRNA expression alterations being due to mRNA degradation from variables such as brain pH and PMI [55]. To control for this potential bias in this study, post-mortem brain tissue from individuals with schizophrenia and non-psychiatric controls were carefully screened and matched for brain pH and PMI in addition to brain hemisphere, age and gender. The tissue pH for pairs 3, 8 and 10 were not as closely matched as the remaining pairs, however there was no correlation between lower pH in the patients, numbers of genes expressed, levels of gene expression on the microarrays or in the relative levels of gene expression measured by real-time PCR. Even though all the variables thought to influence RNA integrity and gene expression were controlled for as much as possible in this study, one cannot rule out the possibility that differences in pH could cause changes in gene expression in post-mortem brain tissue.

In addition to this, another variable which may influence post-mortem tissue gene expression is anti-psychotic medication. The large range of chlorpromazine equivalents (CPE) dosages across the cohort as well as the medication history of one individual being unknown resulted in the inability to perform correlation analyses between anti-psychotic medications and altered gene expression, therefore the influence of anti-psychotic medications on the genes with altered expression in the STG requires further investigation.

## Conclusion

This study has shown altered expression via microarrays and real-time PCR of genes in the STG involved in neurotransmission (particularly glutamate transmission), neurodevelopment, and to a lesser extent presynaptic function. These alterations in gene expression particularly involving glutamate-related transcripts, are likely to have dramatic consequences for neurotransmission in the STG in schizophrenia.

## Methods

### Characteristics of subjects

This study was approved by the University of Newcastle Human Research Ethics Committee, Australia. Blocks of coronally sectioned post-mortem STG tissue (1 cm thick) were sourced from the NSW Tissue Resource Centre, The University of Sydney, Australia. Tissue from 13 pairs of individuals with schizophrenia and controls were matched for gender, age, brain hemisphere, post-mortem interval (PMI) and pH (Table 1). Differences in the age, PMI and pH of the schizophrenia and control groups were not statistically significant (paired 2 tailed t-test). Pairs 1–7 were used for microarray analysis, whilst all 13 pairs were used for the relative real-time PCR studies. These cases were the same cohort used in a previous study of RGS4 expression [31].

### RNA extraction

RNA was extracted as described in [31]. Briefly, a hole punch of grey matter, excluding white matter, was taken from the outer edge of the block of STG from the most caudal coronal brain slice (1 cm thick) containing the STG (Brodmann's Area 22). Total RNA was extracted using TRIzol reagent (Gibco-BRL, USA) as described previously [14]. Briefly, approximately 50–60 mg tissue was added to 1 ml TRIzol reagent and total RNA was prepared according to manufacturer's instructions. RNA integrity was assessed by A260/A280 ratios (> 1.8 and < 2.1) and visualisation of 18s and 28s ribosomal bands by electrophoresis with formaldehyde denaturing 1% agarose gel.

### Microarray procedure

The microarray procedure used in this study was essentially as described in [14]. Briefly, Compugen Version 2 19 K oligonucleotide spotted glass microarrays (Clive and Vera Ramaciotti Centre for Gene Function Analysis and the Adelaide Microarray Facility, Australia) were used to compare the amount of fluorescence (Cy3/Cy5) of each matched pair on a single slide. Matched pairs were analysed in duplicate with a dye swap for the second microarray to reduce the effects of dye incorporation bias. Indirect labeling of the cDNA was performed using the CyScribe Post-Labelling Kit (Amersham Biosciences, UK) as per manufacturer's instructions. Briefly, total RNA (25 μg) was reverse transcribed, using an oligo-dT primer, into cDNA incorporating an amino-allyl modified d-UTP, which was then labelled with either Cy5 or Cy3 fluorescent dyes and hybridised to the microarray for 16 h at 42-C. Post-hybridisation, the microarrays were washed in increasingly stringent solutions of SDS and SSC (1 × SSC and 0.1% SDS at 50°C through to 0.1 × SSC at room temperature) and then dried using centrifugation. Microarrays were scanned with the Axon GenePix 4000B microarray scanner (Molecular Devices, California, USA), the images were then analysed using the GenePix Pro 3.0 software. Areas of the microarray that were affected by background were excluded from further analyses.

### Microarray data analysis

GeneSpring 5.0 (Silicon genetics, USA) was used to normalize per spot and per array (Lowess curve) and to a 50th percentile. To determine the fold change corresponding to a 5% error rate for this microarray platform a sample of control human brain RNA was split in two and labelled with Cy3 and Cy5 and hybridised in duplicate to a glass microarray similar to those used throughout this study. To identify significantly altered genes in the STG, one-class significance analysis of microarrays (SAM) was performed on Log 2 transformed expression data, with 128 permutations using SAM version 2.0 [33].

### Relative real-time PCR

Relative real-time PCR was performed essentially as described previously [32]. Briefly, 100 ng total RNA was treated with DNAse 1 (Invitrogen, USA) and reverse transcribed using oligo dT primers and Superscript II reverse transcriptase (200U/μl) (Invitrogen, USA) as per manufacturer's instructions. Relative Real-time PCR using SYBR green Mastermix (PE Applied Biosystems, UK) and an ABI prism 7900 sequence detection system (PE Applied Biosystems, UK) was performed in triplicate to identify relative changed expression of IPLA2G, PIK31R1, Lin-7b and ATBF1 compared to the housekeeping genes β-actin and 18s rRNA. The statistical significance of the altered expression was tested by a paired two-tailed t-test (p < 0.05), the reported p values were calculated using the relative expression levels compared to 18s RNA. Bivariate correlation analysis was performed to determine if there was any correlation between the altered expression and the age, pH or PMI. Correlation analysis could not be performed for medication as many of the individuals with schizophrenia had large ranges of reported dosage and the medication history of one individual was unknown.

## Authors' contributions

NB carried out all experiments, statistical analysis and drafted the manuscript. RJS assisted with the design of the study, assisted with the supervision of the microarray experiments and helped draft the manuscript. PT conceived and designed the study and helped draft the manuscript. All authors have read and approved the final version of the manuscript.
